# In Situ ATR-FTIR Nonisothermal Kinetic Analysis of Struvite–Dittmarite Thermal Transformation

**DOI:** 10.1021/acsearthspacechem.5c00369

**Published:** 2026-02-16

**Authors:** Sherif Hefney, Damilola Tomi Awotoye, Neal Fairley, Alexander Laskin, Jonas Baltrusaitis

**Affiliations:** † Department of Chemical and Biomolecular Engineering, 1687 Lehigh University, 111 Research Dr., Bethlehem, Pennsylvania 18015, United States; ‡ Casa Software Ltd, Teignmouth TQ14 8NE, U.K.; § Department of Chemistry, Purdue University, West Lafayette, Indiana 47907, United States

**Keywords:** magnesium ammonium phosphate hydrate, struvite, dittmarite, nonisothermal kinetics, in situ ATR-FTIR, linear least-squares spectral analysis

## Abstract

The thermal transformation of magnesium ammonium phosphate hydrates is highly relevant to environmental processes including physicochemical mechanisms associated with nutrient recovery and release. In this study, a nonisothermal kinetic framework was developed using in situ attenuated total reflectance Fourier-transform infrared (ATR-FTIR) spectroscopy to quantitatively describe the struvite (NH_4_MgPO_4_·6H_2_O) to dittmarite (NH_4_MgPO_4_·H_2_O) transformation in the magnesium ammonium phosphate hydrate compound. A linear least-squares (LLS) spectral decomposition approach was applied to temperature-resolved ATR-FTIR data sets to extract the degree of conversion (α) across multiple heating rates. Complementarily, in situ X-ray diffraction (XRD), thermogravimetric analysis, and ex situ Raman spectromicroscopy provided structural and compositional validation of the chemical and crystalline transformations. The resulting kinetics information derived from spectral analysis of phosphate (PO_4_^3–^) and water/hydroxyl/ammonium (H_2_O/OH^–^/NH_4_^+^) vibrational regions identified four thermally distinct stages, corresponding to the release of surface water (adsorbed water), followed by crystalline dehydration associated with struvite to dittmarite transformation, and two subsequent amorphization steps yielding magnesium hydrogen phosphate (MgHPO_4_). Activation energies of 129.3 ± 17.9 and 126.3 ± 16.8 kJ/mol were obtained using the Kissinger analysis for the struvite–dittmarite transformation, while isoconversional Kissinger–Akahira–Sunose (KAS) evaluation indicated an average activation energy of approximately 143.9 ± 5.5 and 140.1 ± 10.9 kJ/mol across multiple α values. These results show that temperature-programmed ATR-FTIR, coupled with LLS spectral analysis, provides a surface-sensitive route for deriving nonisothermal kinetics and identifying coupled structural–vibrational mechanisms in hydrated ammonium phosphate systems.

## Graphical abstract

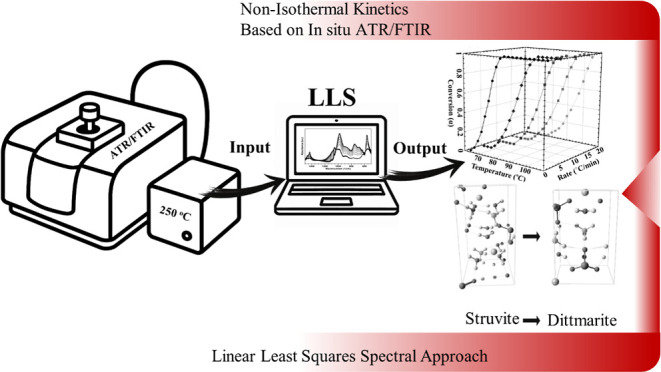

## 1 Introduction

Magnesium ammonium phosphate precipitation is widely regarded as an effective approach for reclaiming nitrogen and phosphorus from wastewater,^1^ yielding a useful and highly crystalline material. Struvite, the most prevalent magnesium ammonium phosphate, is a crystalline solid consisting of a 1:1:1 molar ratio of Mg^2+^, NH^4+^, and PO_4_^3–^, along with six molecules of crystalline water. Hence, struvite (NH_4_MgPO_4_·6H_2_O) is a mineral of high relevance in both environmental and agricultural applications,^2,3^ as it provides essential macronutrients with slow-release properties.^4,5^ Struvite readily precipitates from nutrient-rich wastewater through the magnesium-ammonium-phosphate crystallization process, where the Mg:N:P molar ratio and the solution pH are the primary governing parameters.^6,7^ Complex product speciation results when excess magnesium concentrations, particularly from low solubility MgO or MgCO_3_ minerals, are used.^8^ As a result, a variety of crystalline phases are produced, as evidenced by diverse particle morphologies and Raman spectroscopy measurements.^8^ Struvite and its thermally modified derivatives have also been explored as low-cost sorbent materials for gas capture and energy storage.^9^ Upon heating, struvite progressively releases water and ammonia, leading to mass loss exceeding 50%.^10,11^ At temperatures in the range of 100–150 °C, struvite undergoes partial dehydration, forming dittmarite (MgNH_4_PO_4_·H_2_O) and other intermediate phases that may act as unconventional sorbents for ammonia gas.^9,12^ Hence, recent research has focused on the thermal decomposition mechanisms of struvite to recover valuable magnesium compounds and elucidate its transformation pathways.

Struvite thermal transformations are complex and are closely related to the formation of several magnesium ammonium phosphate phases, particularly dittmarite (MgHPO_4_·H_2_O) and newberyite (MgHPO_4_·3H_2_O).^13–15^ These crystalline phases can nucleate concurrently with struvite during precipitation when pH deviates from the optimal window or when water activity is limited,^12,14^ especially under environmental conditions that favor partial dehydration and/or ammonium loss. Sarkar and Bhuiyan established a systematic framework describing relationships among struvite, dittmarite, and newberyite as functions of heating and hydration states,^12,14,15^ including other relevant phases that can be obtained during the recovery with struvite, such as bobierrite (Mg_3_(PO_4_)_2_·8H_2_O).^16^ Graeser et al. reported natural K-struvite from the Rossblei deposit in Austria containing newberyite,^17^ while Perwitasari et al. detected newberyite under ferric ion contamination at a pH value of 7.5.^18^ Sarkar investigated magnesium ammonium phosphate transformations using thermogravimetric analysis (TGA) and reported an initial thermal conversion at roughly 55 °C under air.^12^ The total mass loss was around 50%, consistent with NH_3_ volatilization and the release of six crystalline water molecules; below 50 °C, only physisorbed water was removed.^12^ In an excess-water environment, dehydration proceeded primarily through loss of coordinated water to yield dittmarite, which remained thermally stable until a principal decomposition near 221 °C.^12^ Zhang et al. reported that KMgPO_4_·6H_2_O dehydration began around 60 °C and was completed by ∼250 °C.^19^ Differential thermal analysis (DTA) showed a single prominent endotherm at 105 °C, indicating a one-step release of all six waters. The corresponding mass loss of ∼58.5% remained invariant across heating rates of 2–20 K/min. Isoconversional kinetics showed activation energies in the range of 86 to 106 kJ/mol.^19^ Wu et al. studied dittmarite using TGA and observed two decomposition stages: an initial step at 80–187 °C followed by a higher-temperature stage up to ∼700 °C,^20^ with activation energies of 147.4 and 212.7 kJ/mol, respectively.^20^ Struvite fully decomposes into amorphous MgHPO_4_ at 250 °C, with a mass loss of 51%, as similarly confirmed by TGA measurements^15^ via reaction 1:

1 $$MgNH_{4}PO_{4}\cdot 6H_{2}O\overset{heat}{\to }MgHPO_{4}+NH_{3}+6H_{2}O$$

Importantly, the heating rate influenced the observed transformation temperatures, with higher heating rates shifting decomposition to higher values. The maximum reaction rate for synthetic struvite decomposition increased from approximately 104 °C at 1 °C/min to about 190 °C at 20 °C/min, with activation energies reported in the range 46–84 kJ/mol. X-ray diffraction (XRD) measurements further indicated a loss of crystallinity at around 110 °C.^15^ Dittmarite also exhibited a single endothermic reaction with a total mass loss of 20% via reaction 2:

2 $$MgNH_{4}PO_{4}\cdot H_{2}O\overset{heat}{\to }MgHPO_{4}+{NH}_{3}+H_{2}O$$

An infrared spectroscopy-based approach can provide a chemical-selection-driven thermal kinetic framework for thermally activated transformations. However, its application to the magnesium ammonium phosphate hydrate system has never been reported. Pannico et al. obtained kinetic data using time-resolved Fourier transform infrared (FTIR) measurements to investigate the thermal decomposition of epoxy resins.^21^ Data were collected under isothermal conditions at 180 °C in a controlled gas environment in transmission mode, enabling precise monitoring of absorbance changes for specific functional groups over time.^21^ Degradation kinetics were quantified by tracking peak intensities and integrated band areas corresponding to aromatic rings, alkyl chains, and carbonyl groups. The resulting conversion curves enabled the extraction of rate constants and revealed multiple degradation pathways with distinct reaction rates.^21^ Li et al. developed a kinetic analysis approach for thermal transformations using in situ ATR-FTIR spectroscopy.^22^ They tracked the temperature-dependent changes in characteristic absorption bands particularly the oxazine ring at 940 cm^–1^ and calculated the degree of conversion (α) using the Lambert–Beer law.^22^ Multiple heating rates were applied and the conversion-temperature data were fitted to established nonisothermal kinetic models, enabling the extraction of the activation energies and reaction rate equations. The strong agreement with conventional kinetic analyses demonstrated that FTIR offers enhanced resolution and mechanistic insights for thermal transformation studies,^22^ suggesting that a similar kinetic framework can be developed for magnesium ammonium phosphate thermal transformations.

While previous studies have focused on the decomposition of pure struvite using conventional bulk thermal analysis methods, the present study focuses on the kinetic analysis of a complex system of magnesium ammonium phosphate hydrates using a combination of linear least-squares (LLS) and in situ ATR-FTIR. Specifically, using the LLS fit of the evolving spectral data, reaction progress was quantified. Reactant and product spectra in temperature-resolved experiments were used to obtain the degree of conversion (α). This allowed for a deconvolution of reactant and product contributions, therefore providing molecular-level insights. Complementary in situ structural analyses were employed to follow the lattice evolution and track associated compositional changes at the surface during heating. In addition, chemical imaging using Raman spectromicroscopy coupled with true component analysis was used to resolve and spatially map the spectral signatures of the constituent phases.

## 2 Methodology

### 2.1 In Situ XRD and Structure Refinement of the Magnesium Ammonium Phosphate Hydrate Reaction Products

Commercial magnesium ammonium phosphate hydrate was used (Sigma-Aldrich, 99.997% trace metals basis) with a molecular weight of 137.31 g/mol and chemical formula of MgNH_4_PO_4_·*x*H_2_O. In situ XRD was carried out on a PANalytical Empyrean diffractometer using Cu Kα radiation (λ = 1.5406 Å) with an Anton Paar DHS-1100 hot stage. Scans were collected in air while the temperature was stepped from 25 to 250 °C with a heating rate of 10 °C/min under a carbon dome. Phase analysis and Rietveld refinement were conducted in Profex (BGMN).^23^ In refinement, the standard files from the International Centre for Diffraction Data (ICDD) were used for struvite (01-075-0674) and newberyite (00-020-0663).^24,25^ The dittmarite structure was based on the Crystallography Open Database (COD, 9012526),^26^ replacing Mn from the original structure with Mg, in addition to ensuring the matching of lattice parameters and diffraction pattern with previous reports.^27,28^

### 2.2 Temperature-Programmed In Situ Total Reflectance Fourier Transform Infrared Spectroscopy (ATR-FTIR)

Magnesium ammonium phosphate samples were analyzed on a PIKE GladiATR heated ATR stage (calibrated based on melting point of benzoic acid) coupled to a Thermo Nicolet iS50 FTIR. Spectra were acquired over a 4000–500 cm^–1^ wavenumber range. For each spectrum, 16 scans were averaged at 4 cm^–1^ resolution with a data spacing of 0.482 cm^–1^ resolution using a DTGS detector and a diamond single-bounce ATR crystal. A typical experiment was performed using TemPro7 software in air as follows: (i) a background spectrum was recorded of the clean crystal at all temperatures from 25 to 250 °C every 2.5 °C at the given temperature ramp rate and (ii) at the same temperature ramp rate, magnesium ammonium phosphate sample spectra were collected every 2.5 °C. The collected spectra were then background-subtracted and used in kinetic analysis.

The in situ ATR-FTIR background-subtracted spectra collected at various heating rates were processed using the Linear Analysis module as implemented in CasaXPS.^29^ Specifically, LLS was applied to decompose each experimental ATR-FTIR spectrum as a linear combination of selected reference spectra representing distinct chemical vibrational regions or transformation stages. This procedure fitted each spectrum into the weighted contributions normalized to unity, which provided the temperature-dependent conversion fraction (α) for every heating rate. Specifically, a set of three representative spectra was used as the basis, namely, acquired at 25 °C (initial spectrum), 100–125 °C (intermediate spectrum or after the first transformation), and 250 °C (final observed transformation and spectrum). The linear combination is described by eqs 3 and 4 given *m* linearly independent functions^30^

3 $$y\left(x\right)=c_{1}f_{1}\left(x\right)+c_{2}f_{2}\left(x\right)+\cdot \cdot \cdot \cdot +c_{m}\cdot f_{m}\left(x\right)$$

4 $$y_{\exp}\left(x\right)=y\left(x\right)+r\left(x\right)$$

where *y*(*x*) is the constructed spectrum, *r*(*x*) is the residual, and *f*_1_(*x*), *f*_2_(*x*), and *f*_*m*_(*x*) are reference spectra at various temperatures. *c*_1_, *c*_2_, and *c*_m_ are the contribution coefficients (weights) of each basis spectrum *f*_*i*_(*x*). α is then determined according to eqs 5 and 6, where ${\mathrm{c̅}}_{i}$ is the normalized contribution coefficient:

5 $${\mathrm{c̅}}_{i}=\frac{c_{i}}{\sum_{i=1}^{m}c_{i}}$$

If *c*_1_ is the weight of *f*_1_(*x*) which is the basis at room temperature (before any reaction), then α is obtained via^6^

6 $$\alpha =1-{\mathrm{c̅}}_{1}$$

For every spectrum acquired during heating, the algorithm determined the optimal set of scalar coefficients (contributions or weights) that minimized the squared difference between the measured spectrum and its reconstruction from the basis set. The summation of these weights was normalized and therefore equal to unity ( ${\mathrm{c̅}}_{1}+{\mathrm{c̅}}_{2}+{\mathrm{c̅}}_{3}=1$ ). This approach allowed tracking the evolution of spectral vibrational regions quantitatively, and when plotted against temperature or time, the contributions can be interpreted as conversion-like parameters used for kinetic modeling. The term basis is appropriate in this context as the selected spectra collectively span the space of observed transformation states, analogous to basis sets spanning function spaces in mathematical decomposition frameworks. The converted spectral data were then analyzed using both the Kissinger and Kissinger–Akahira–Sunose (KAS) methods to extract the activation energy of struvite–dittmarite transformation.

#### 2.2.1 Kissinger Kinetics Method for Determining Magnesium Ammonium Phosphate Hydrate Transformation Activation Energy

It is assumed that the magnesium ammonium phosphate thermal transformation proceeds through a series of reactions and that the effective reaction rate depends on temperature and the degree of conversion, as described by eq 7:

7 $$\frac{d\alpha }{dt}=k\left(T\right)f\left(\alpha \right)$$

where

8 $$k\left(T\right)={Ae}^{-E_{a}/RT}$$

Here, α denotes the degree of conversion (derived from the LLS analysis), where α = 0 corresponds to the start and α = 1 corresponds to the completion of the transformation. *T* is the absolute temperature, *t* is the time, *f*(α) is the reaction rate expression, *k*(*T*) is the Arrhenius rate constant, and *E*_a_, *A*, and *R* are the activation energy, pre-exponential factor, and ideal gas constant, respectively. Under nonisothermal conditions, constant heating rate (β) is defined by eq 9:

9 $$\beta =\frac{dT}{dt}$$

At a fixed β, the rate $\frac{d\alpha }{dt}$ rises with *T* and reaches a maximum at a temperature value (*T*_p_), which satisfies eq 10:

10 $$\frac{d}{dt}{\left(\frac{d\alpha }{dt}\right)|}_{T=T_{p}}=0$$

This condition leads to eq 11

11 $$\ln\left(\frac{\beta }{T_{p}^{2}}\right)=\ln\left(\frac{AR}{E_{a}}\right)-\frac{E_{a}}{RT_{p}}+\ln\left(-f^{\prime }\left(\alpha_{p}\right)\right)$$

According to the Kissinger assumption, near the peak region, the conversion α_p_ is nearly constant across heating rates, therefore, *f*(α_p_) can be considered constant. That yields the practical Kissinger eq 12:^31^

12 $$\ln\left(\frac{\beta }{T_{p}^{2}}\right)=\ln\left(\frac{AR}{E_{a}}\right)-\frac{E_{a}}{RT_{p}}$$

where the activation energy *E*_a_ is determined from the temperature *T*_p_ corresponding to the maximum reaction rate at each heating rate (β). The slope of the plot of $\frac{1}{T_{p}}$ versus $\ln\left(\frac{\beta }{T_{p}^{2}}\right)$ yields $\frac{{-E}_{a}}{R}$ , and *E*_a_ for a specific magnesium ammonium phosphate thermal transformation can be calculated using a series of nonisothermal ATR-FTIR spectra.

#### 2.2.2 Kissinger–Akahira–Sunose (KAS) Isoconversional Method for Evaluating α-Dependent Activation Energies

The classical Kissinger analysis uses only the peak temperature *T*_p_ which satisfies ( $\frac{d^{2}\alpha }{dt^{2}}=0$ ) measured at several heating rates β to obtain a single apparent activation energy that represents the main event and implicitly assumes one dominant step. In KAS, the activation energy depends on α, therefore it can provide a profile of activation energies and an average value. These barriers can be obtained for the thermal transformation without presuming a reaction model.

Equation 13 can be obtained by substituting eq 7 into [Disp-formula eq8] and applying integration, where $dt=\frac{dT}{\beta }$ , the integral form under linear heating (β is constant^31^) is

13 $$g\left(\alpha \right)=\int_{0}^{\alpha }\frac{d\alpha }{df\left(\alpha \right)}=\int_{0}^{t}k\left(T\right)dt=\frac{A}{\beta }\int_{0}^{T_{\alpha }}e^{-E_{\alpha }/RT_{\alpha }}dT$$

where *E*_α_ is the activation energy at a specific α. This leads to the KAS eq 14:

14 $$\ln\left(\frac{\beta }{T_{\alpha }^{2}}\right)=\ln\left(\frac{AR}{E_{\alpha }}\right)-\frac{E_{\alpha }}{RT_{\alpha }}-\ln g\left(\alpha \right)$$

For a fixed conversion α across several β values (isoconversional principle), *g*(α) is a constant. Approximating the temperature integral (Murray–White/Starink class) leads to a linear relation where $\ln\left(\frac{AR}{E_{\alpha }}\right)-\ln\;g\left(\alpha \right)$ collapses into a single constant intercept (for that α). Thus, a linear KAS^15^ relation can be obtained:^31^

15 $$\ln\left(\frac{\beta }{T_{\alpha }^{2}}\right)=constant-\frac{E_{\alpha }}{RT_{\alpha }}$$

The slope of $\frac{1}{T_{\alpha }}$ versus $\ln\left(\frac{\beta }{T_{\alpha }^{2}}\right)$ provides $\frac{{-E}_{a}}{R}$ . This approach allows for identification of variations in activation energy across the reaction progress. In contrast, the Kissinger method gives a single apparent activation energy for the whole reaction (assuming a simple single-step reaction).

### 2.3 Ex Situ Raman Spectrophotometry of Magnesium Ammonium Phosphate Microparticles via Hyperspectral Analysis

Raman spectra were collected on a WITec alpha300R confocal microscope (532 nm excitation, Zeiss 20*x*/0.4 objective, G2:600 g mm^–1^ grating), over 200–4000 cm^–1^ (centered at 2299 cm^–1^). The laser power of the sample was approximately 54 mW, and the spectrometer was calibrated to the Si band before each run. For single-point measurements, an individual particle was selected under the microscope, its position was fiducially marked on the glass slide, and the slide was heated ex situ on a hot plate with the temperature monitored by an IR thermometer. Once the target temperature was reached, the slide was returned to the Raman instrument stage, the same particle location was positioned, and both an optical image and a spectrum were recorded. For mapping, temperature-programmed samples were prepared using the same heating system as the FTIR experiments with the heating rate of 10 °C min^–1^. At each designated temperature, the specimen was mapped over a scan width × scan height of 450.100 μm × 283.400 μm with a 5s integration time per spectrum.

Raman data processing and multivariate analysis employed true component analysis (TCA) in WITec Project 5.1 to visualize and differentiate the spectral contributions of the constituent phases. Two-dimensional spectral maps were acquired along the *x*–*y* plane to assess the lateral compositional variations across the crystal surface. The TCA algorithm was employed to construct spectral intensity distribution images for the distinct spectral components. In TCA, each spectrum $\vec{S_{i}}$ from the hyperspectral data set is expressed as a linear combination of basis spectra (eq 16) according to^32^

16 $$\vec{S_{i}}=\mathrm{B̂}\vec{H_{i}}+\vec{E_{i}}$$

where *B̂* is the matrix of basis spectra, $\vec{H_{i}}$ represents the mixing values for the spectrum *i*, and $\vec{E_{i}}$ is the residual (error) spectrum. The optimal mixing values are obtained by least-squares minimization in eq 17:^32^

17 $${\left(\vec{S_{i}}-\mathrm{B̂}\vec{H_{i}}\right)}^{2}=minimum$$

### 2.4 Thermal Analysis Based on Differential Scanning Calorimetry and Thermogravimetric Analysis (DSC-TGA)

The TGA measurements were carried out on a PerkinElmer Pyris instrument using approximately 10 mg by heating from 40 to 400 °C at rates of 5 and 10 °C/min under a nitrogen purge of 20 mL/min. Each run began with a 1 min hold at 40 °C to stabilize the baseline. Complementary DSC measurements were conducted on a TA Instruments DSC Q2000 (V24.10 Build 122) instrument equipped with a standard RC cell and nitrogen purge gas. Approximately 10 mg of sample was nonsealed in aluminum hermetic pans and heated from 25 to 400 °C at a heating rate of 10 °C/min.

### 2.5 Quantum Chemical Calculations

All first-principles calculations were performed using the CRYSTAL23 code, which uses a periodic ab initio approach based on localized Gaussian-type orbitals within the Kohn–Sham density functional theory (DFT).^33^ The BECKE 88-[LEE-YANG-PARR] (B3LYP) hybrid functional was used to describe electron exchange and correlation effects, and all-electron Gaussian basis sets optimized for solid-state systems were applied. Reciprocal-space integration utilized a Monkhorst–Pack grid (6 × 6 × 6 with 64 *k*-points in the irreducible Brillouin zone), ensuring total-energy convergence to within 10^–11^ Ha. The SCF convergence threshold for the energy change was fixed at 1.0 × 10^–7^ Ha. In addition, dispersion interactions were treated using the DFT-D3­(BJ) correction scheme of the Grimme method.^34,35^ The basis sets were 8-411G­(d) for oxygen, 31G­(p) for hydrogen, 8-511G­(d) for magnesium, 85-21G­(d) for phosphorus, and 6-31G­(d) for nitrogen.^36–40^ Vibrational frequencies were computed at the Γ point within the harmonic approximation. The dynamic matrix (Hessian), which contains the second derivatives of the total energy with respect to atomic displacements, was obtained numerically from analytical first-derivative gradients. The resulting mass-weighted dynamic matrix was diagonalized to yield eigenvalues (squared angular frequencies) and eigenvectors (normal modes). Infrared (IR) intensities were evaluated within the same harmonic framework using the coupled-perturbed Kohn–Sham (CPKS) method as implemented in CRYSTAL23. The IR intensity of each vibrational mode is proportional to the square of the derivative of the macroscopic dipole moment with respect to its normal coordinate.^41^ Dipole derivatives were obtained from the Born effective charge tensors, which were computed by using the Berry-phase approach. These tensors quantify the polarization change per atomic displacement and thus determine the IR activity. All calculations were carried out under periodic boundary conditions at 0 K. Only atomic parameters were optimized by using lattice parameters obtained from refined crystal structures before the vibrational analysis to ensure a stationary point on the potential-energy surface.

### 2.6 Safety Statement

No unexpected or unusually high safety hazards were encountered during the reported work. All experimental procedures, including in situ XRD, ATR-FTIR spectroscopy, and Raman measurements, were performed by following standard laboratory safety protocols. Proper personal protective equipment (PPE) was used, and appropriate safety measures for high-temperature heating and laser operations were strictly maintained.

## 3 Results and Discussion

### 3.1 In Situ XRD Phase Composition Analysis

Figure 1a shows the evolution of XRD patterns of commercial magnesium ammonium phosphate hydrate as a function of the temperature. Between 25 and 100 °C, the XRD patterns reveal a mixture of crystalline phases with struvite as the dominant phase. Struvite is identified by its well-established reflections at 14.9° (101), 15.7 (002), 16.4° (011), and 20.8° (111), consistent with previously reported diffraction patterns (PDF#71-2089).^42–44^ In contrast, dittmarite is detected primarily through its strongest peak at 10.0°, corresponding to the (010) plane. At 125 °C, struvite peaks disappear, while those of dittmarite and newberyite emerge. By 150 °C, a complete transformation to the dittmarite phase is evident, as confirmed by the loss of all other peaks and stabilization of dittmarite reflections. Newberyite formation, although minor, is indicated by small peaks at 19.7° and 34.6°, assigned to the (102) and (041) planes, respectively (PDF#70-2345).^45^ These observations indicate that while struvite remains the major phase at lower temperatures, newberyite forms as a transient phase.

**Figure 1:**
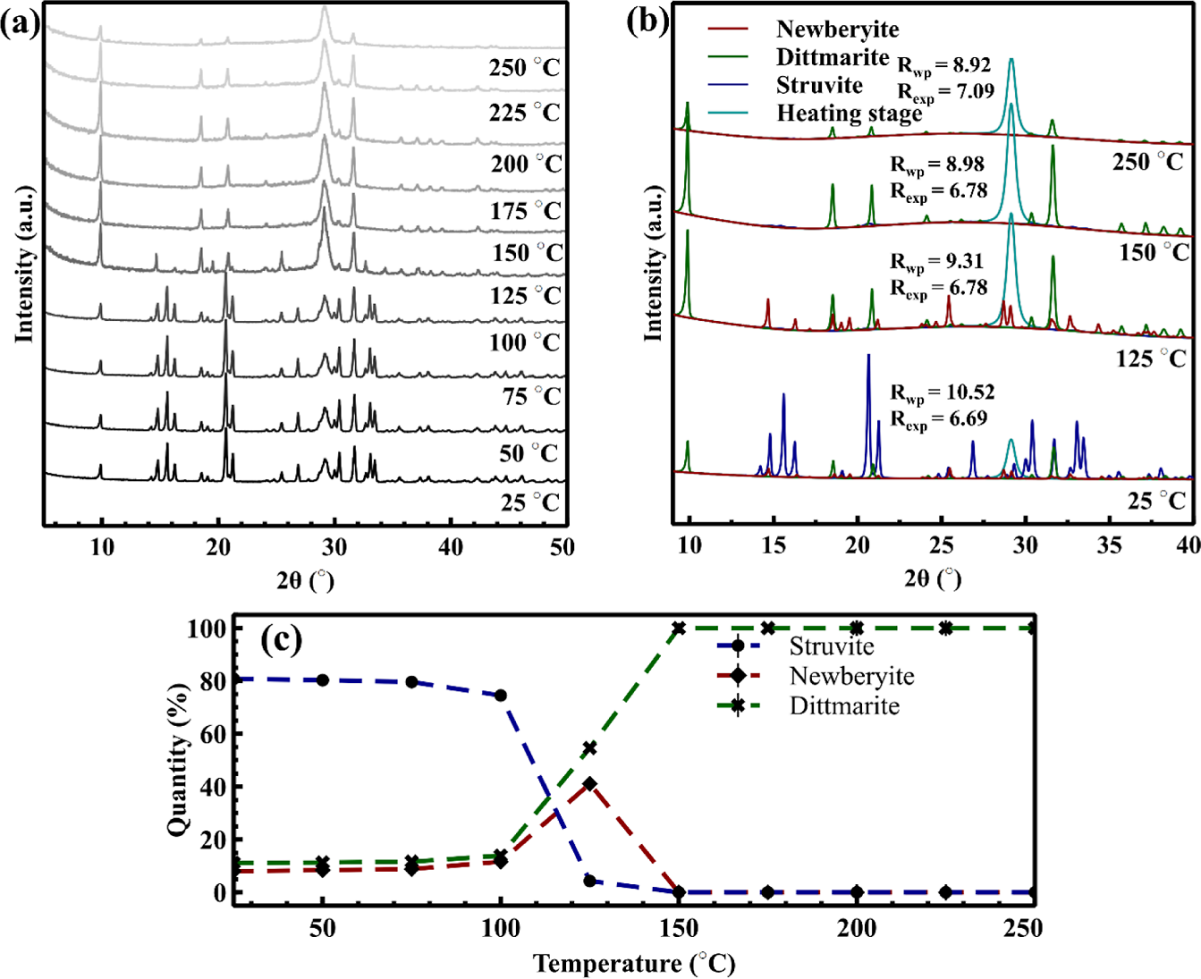
(a) In situ XRD patterns of magnesium ammonium phosphate hydrate from 25 to 250 °C with a heating rate of 10 °C/min. (b) Phase identification and refinement confirming transformation from struvite (blue) to dittmarite (green) and trace newberyite (red). (c) Phase quantification using Rietveld refinement showing struvite depletion and dittmarite formation with increasing temperature as obtained via in situ XRD.

The Rietveld refinement shown in Figure 1b further quantifies the phase distribution by fitting the experimental patterns with reference crystalline structures. At 25 °C, the fitting requires a combination of struvite, dittmarite, and newberyite, indicating a complex initial phase composition. Above 125 °C, only the dittmarite phase is detected, indicating a complete conversion of struvite to dittmarite. The quantitative phase evolution, shown in Figure 1c, supports this transformation: struvite content decreases sharply from 100 to 125 °C, while dittmarite increases correspondingly, and newberyite appears only transiently, reaching a maximum around 125 °C before disappearing at higher temperatures. Table 1 provides detailed temperature-resolved refined lattice parameters and apparent crystallite sizes (D). The struvite unit cell parameters remain relatively unchanged from 25 to 100 °C, with typical values of *a* = 0.695–0.696 nm, *b* = 0.614–0.615 nm, and *c* = 1.122–1.123 nm. Meanwhile, the apparent D along the (111) direction increases from 246 to 465 nm, indicating mild coarsening rather than fragmentation. It is important to note that crystallite size obtained from XRD corresponds to the average distance over which lattice planes with Miller indices (*hkl*) scatter in phase, maintaining a uniform *d*-spacing and orientation. Therefore, it does not necessarily reflect the physical particle size. The observed increase can be attributed to thermal annealing in the early stage heating regime, where enhanced atomic mobility relieves internal strain and reduces crystal defects, promoting the formation of larger coherent domains prior to decomposition. Above 125–150 °C, the diffraction peaks of struvite disappear as dittmarite becomes the dominant phase. Dittmarite exhibits continued crystallite growth with temperature, reaching 634 nm at 200 °C. Newberyite, although clearly resolved at 125 °C, shows irregular crystallite sizes up to 853 nm, suggesting rapid growth, followed by decomposition, consistent with its transient or metastable nature. A similar temperature-dependent growth behavior is observed for dittmarite, which coincides with the onset of amorphous phase formation. Literature reports indicate that amorphous magnesium phosphate begins to form near 110 °C, while dittmarite decomposition initiates ∼at 221 °C, with complete amorphization occurring around 250–350 °C.^12,15^

**Table 1:** Refined Lattice Parameters (*a*, *b*, and *c*) and Apparent Crystallite Sizes (*D*) of Struvite, Dittmarite, and Newberyite Phases Obtained from In Situ XRD Rietveld Refinement at Different Temperatures^a^

temperature (°C)	struvite (nm)				dittmarite (nm)				newberyite (nm)				statistics	
	*a*	*b*	*c*	*D*_111_	*a*	*b*	*c*	*D*_010_	*a*	*b*	*c*	*D*_111_	*x* ^2^	GoF
25	0.695	0.614	1.122	246	0.562	0.877	0.479	448	1.023	1.069	1.001	184	2.4	1.5
50	0.695	0.614	1.122	261	0.562	0.877	0.479	274	1.022	1.071	1.002	155	2.4	1.5
75	0.696	0.615	1.123	275	0.563	0.877	0.480	292	1.022	1.073	1.002	141	2.2	1.4
100	0.695	0.615	1.123	465	0.562	0.877	0.480	314	1.023	1.074	1.003	139	1.9	1.3
125	-	0.563	0.878	0.481	217	1.023	1.076	1.003	853	1.9	1.3			
150	-	0.563	0.878	0.481	318	-	1.7	1.3						
200	-	0.564	0.878	0.482	634	-	1.6	1.2						

a *D* represents the apparent crystallite size along the specified crystallographic plane; *x* ^2^ and GoF denote the overall quality of refinement and the goodness of fit statistics.

### 3.2 In Situ ATR-FTIR Spectroscopy and Vibration-Specific Spectral Analysis

ATR-FTIR spectra obtained during magnesium ammonium phosphate hydrate heating from 25 to 250 °C at 2.5 °C/min are shown in Figure 2a. The spectra can be divided into chemically meaningful vibrational regions to allow for the functional group-resolved kinetic analysis. As shown in Figure 2b, the vibrational region between 1300 and 800 cm^–1^ corresponds to P–O vibrations in PO_4_^3–^, particularly the asymmetric and symmetric stretching modes of phosphate groups.^46^ Around 85–100 °C, depending on the heating rate, the evolution of PO_4_^3–^ peaks resulted in a prominent doublet of nearly equal intensity. When the temperature reaches 250 °C, the peak broadens, which might be attributed to an amorphous magnesium phosphate structure. Another important spectral region lies between 3600 and 2800 cm^–1^ and includes O–H and N–H stretching vibrations from water, any residual hydroxyl ions and ammonium ions.^47^ This vibrational region exhibited a systematic loss of intensity with temperature, signifying the progressive loss of hydrogen bonding, which is consistent with dehydration and NH_4_^+^ loss. This was further supported by decreasing bands at approximately 1430 cm^–1^/1693 cm^–1^ (N–H and H_2_O bending).^48,49^

**Figure 2:**
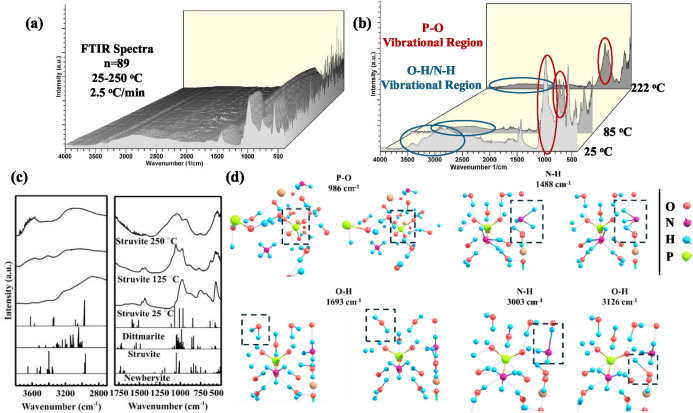
(a) 3D stack of 226 in situ ATR-FTIR spectra collected during magnesium ammonium phosphate heating from 25 to 250 °C at 2.5 °C/min. (b) Spectral vibrational regions of interest containing the PO_4_^3–^ vibrational region at ∼1000 cm^–1^ and O–H/N–H vibrational region at ∼3000 cm^–1^ that were used in kinetic analysis. (c) Temperature-resolved FTIR spectra of magnesium ammonium phosphate at 25, 125, and 250 °C compared to DFT-simulated spectra for struvite, dittmarite, and newberyite. (d) Selected IR vibrations based on DFT calculations of struvite, dittmarite, and newberyite.

Overall, spectral assignments of phosphate-containing materials are complex.^13,32,48–50^ To aid the assignments, IR spectra were calculated by using periodic boundary DFT combined with a Gaussian basis set and dispersion corrected B3LYP hybrid density functional. The calculated discrete vibrations of struvite, dittmarite, and newberyite are shown in Figure 2c, with the specific vibrational mode of interest shown in Figure 2d. The DFT calculations revealed that newberyite has a peak at 1049 cm^–1^, while struvite exhibits several peaks between 947 and 1100 cm^–1^. This supports a broad PO_4_^3–^ band assignment in struvite, while a sharp peak for newberyite was observed in experimental spectra. Notable was the absence of peaks in the range 1450–1600 cm^–1^ in newberyite. On the other hand, dittmarite exhibited four peaks, three of them with higher intensity at 967, 1009, and 1077 cm^–1^ in addition to a lower intensity peak at 1118 cm^–1^. This is comparable to the bands that occur in the experimental spectrum at 125 °C. In Figure 2, the intense vibration of PO_4_^3–^ was observed at 986 cm^–1^. Although the free PO_4_^3–^ ion belongs to the *T*_d_ point group, its symmetry is reduced to *C*_s_ site symmetry and *C*_2v_ factor group symmetry within the struvite/dittmarite-type crystal (space group *Pmn*2_1_ and point group *mm*2), as previously reported.^46^ This reduction gives rise to vibrations belonging to *A*_1_, *B*_1_, and *B*_2_ irreducible representations in the crystal spectra. It can be noticed that this splitting effect is clearer in dittmarite than in struvite due to higher distortion.^51^ At 1693, 3003, and 3126 cm^–1^, water and NH_4_^+^ exhibit peaks in the calculated spectrum closely matching experimental spectral features.

A detailed analysis of the PO_4_^3–^ vibrational region between 1500 and 500 cm^–1^ is shown in Figure 3 and indicates that increasing temperature leads to a red shift in the phosphate band near 1000 cm^–1^. This shift results from a decrease in the band at ∼973 cm^–1^ accompanied by the growth of a shoulder near ∼1050 cm^–1^. Therefore, the observed frequency shift arises from this gradual redistribution of the band intensities, which can be analyzed to extract additional mechanistic and kinetic information. The band at 973 cm^–1^ is attributed to *v*_3_(PO_4_^3–^) in struvite. Also, the *v*_4_(O–P–O) bending mode of PO_4_^3–^ and vibrational modes of water appear at 567 cm^–1^ and 757/881 cm^–1^, respectively.^50^ The intensity loss of these bands with increasing temperature is consistent with the loss of crystalline water, which causes PO_4_^3–^ distortion and therefore lowers degeneracy. In an ideal tetrahedral (*T*_*d*_) environment, the free PO_4_^3–^ anion exhibits four vibrational modes: *v*_1_ (*A*_1_) symmetric PO_4_^3–^ stretch, *v*_2_ (E) symmetric O–P–O bend, and *v*_3_,*v*_4_ (F_2_) asymmetric stretching and bending.^46,48^ All are Raman-active, whereas only *v*_3_ and *v*_4_ are IR-active.^50^ When the anion occupies a lower-symmetry lattice site, degeneracies are removed, and modes that are IR-forbidden vibrations become allowed. Consequently, *v*_3_,*v*_4_ (F_2_) vibrations split into five active IR bands and one inactive mode (*A*_2_, according to the *C*_2*v*_ character table),^46,52^ and *v*_2_ (E) splits into four bands, three of which are IR active. Factor-group (correlation field), and interactions within a crystal may further split each mode into multiple components, for example, *v*_1_ (*A*_1_) mode splits into two bands, *A*_1_ and *B*_2_.^46^ Similar symmetry considerations apply to NH_4_^+^, which also belongs to the *T*_d_ point group. Two main vibrational modes of NH_4_^+^ are detected at 1429 and 1468 cm^–1^ and correspond to asymmetric bending vibrations *v*_4_(NH_4_^+^).^51^ This pair arises from the splitting of the *v*_4_ (F_2_) mode due to symmetry reduction in the crystalline environment.

**Figure 3:**
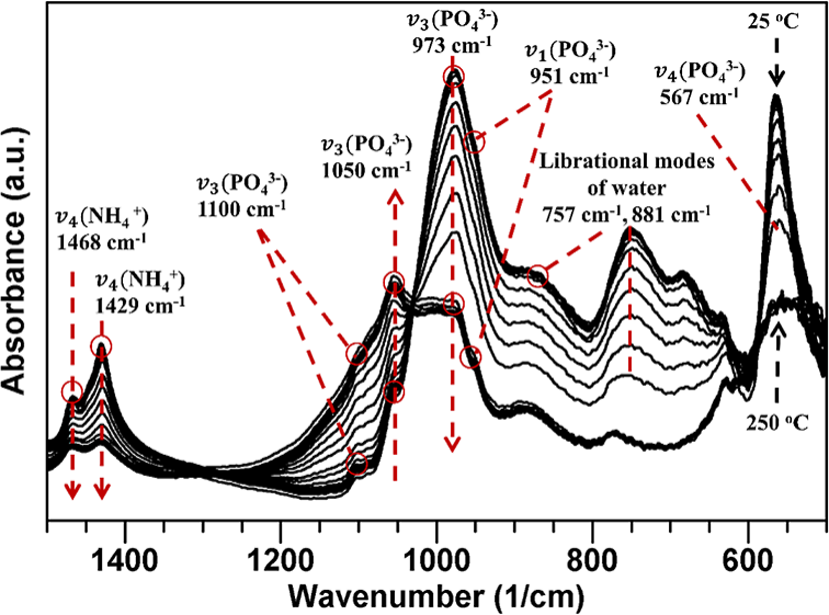
In situ ATR spectra for temperature-dependent evolution of the magnesium ammonium between 1500 and 500 cm^–1^, heating from 25 to 250 °C.

### 3.3 Nonisothermal Conversion Kinetics

In situ ATR-FTIR spectroscopy was employed to investigate the thermally induced transformation of magnesium phosphate using varying heating rates (1, 5, 10, 15, and 20 °C/min). The analysis focused on the specific vibrational regions discussed in Figure 2b to isolate and characterize the corresponding structural and chemical changes. This information is generally not accessible from bulk thermal analysis such as TGA or DSC. As shown in Figure 4a, the PO_4_^3–^ vibrational region (transformations II) near 1000 cm^–1^ exhibits a well-defined thermal transformation profile between ∼65 and 110 °C, with onset and completion temperatures shifting as a function of heating rate. At slower heating rates (1 and 5 °C/min), the transformation begins earlier (65–70 °C) and completes by 84.5–97 °C. At higher heating rates (15–20 °C/min), the reaction initiates at ∼75–80 °C and extends up to ∼107–110 °C. This kinetic shift is characteristic of thermally activated processes and confirms a distinct reaction pathway driven by increasing temperature.

**Figure 4:**
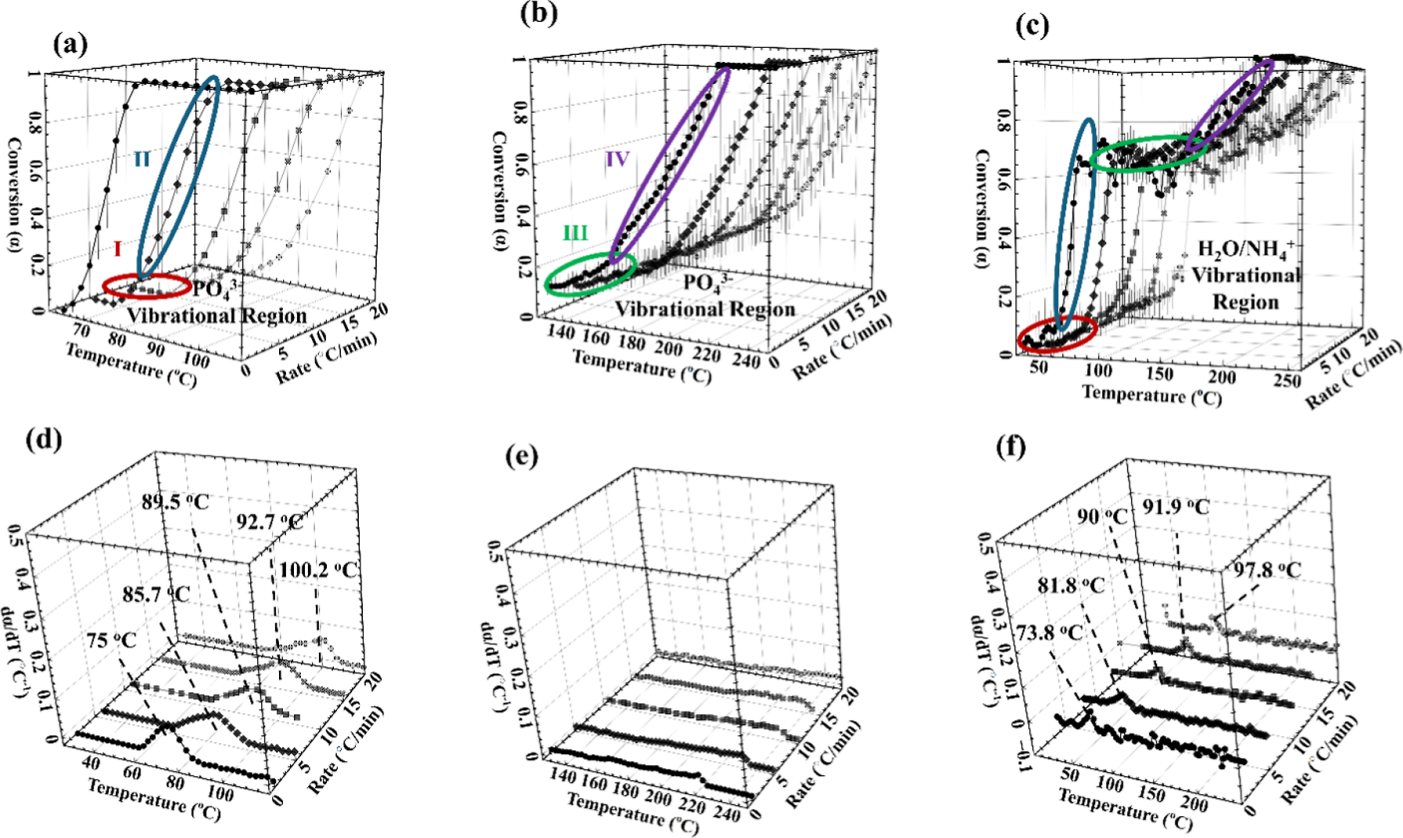
3D plots of conversion (α) and the temperature derivative (dα/d*T*) for selected ATR-FTIR spectral vibrational regions as a function of temperature and heating rate. α vs temperature for the PO_4_^3–^ vibrational region (a) from 25 to 125 °C and (b) from 125 to 250 °C, where thermal transformations are color coded, and (c) H_2_O/NH_4_^+^ vibrational region from 25 to 250 °C. (d–f) dα/d*T* vs temperature showing rates and peaks of transformations.

Figure 4b shows that the PO_4_^3–^ vibrational region undergoes two additional transformation regimes at higher temperatures, designated as III and IV. Transformation III occurs between ∼150 and 200 °C (beginning at ∼150 °C for 1 °C/min and shifting toward ∼200 °C at 20 °C/min). Transformation IV begins between 150 and 200 °C and extends to ∼200–250 °C. Transformation III exhibits minimal sensitivity to the heating rate. In contrast, transformation IV exhibits a heating rate dependence, indicating a more strongly thermally activated pathway. The elevated error margins in transformations III and IV limit the reliability of the quantitative analysis. These two high-temperature regimes (transformations III and IV) likely correspond to the stepwise removal of residual structural water and ammonia, ultimately yielding an amorphous or poorly crystalline magnesium phosphate phase, consistent with TGA/DSC observations (vide infra). The first transformation (II, up to ∼110 °C) is well-supported in the literature and corresponds to the conversion of struvite (MgNH_4_PO_4_·6H_2_O) into dittmarite (MgNH_4_PO_4_·H_2_O) through the loss of five coordinated water molecules.^6^ Because these water molecules participate in the hydrogen-bonding network surrounding the phosphate groups, their removal leads to a distinct reorganization in the PO_4_^3–^ vibrational region, consistent with the observations in Figure 3. Transformations III and IV reflect further dehydration and ammonia release, producing an amorphous magnesium phosphate phase, as described in eq 2. This transformation IV even reported for Na-struvite crystals and interpreted to the loss of crystalline water with formation of NaMgPO_4_.^53^

The evolution of the degree of conversion with temperature in Figure 4c for the H_2_O/NH_4_^+^ stretching vibrational region (2800–3600 cm^–1^) reflects transformations involving molecular water and ammonium ions within magnesium ammonium phosphate. The same four transformation regimes are observed in Figure 4c. The first occurs from room temperature to ∼65 °C and is unique to this (H_2_O/NH_4_^+^) vibrational region. It can be attributed to the release of physically adsorbed or surface-bound water associated with noncrystalline hydration external to the phosphate framework. The second, third, and fourth transformations (65–110 °C, depending on the heating rate, and >110 °C, respectively) aligned with the changes observed in the PO_4_^3–^ vibrational region (Figure 4b) and are therefore linked to the progressive loss of coordinated (structural) water and ammonium. Their synchronized behavior in both vibrational domains indicates a coupled process, supporting the interpretation that structural dehydration (struvite to dittmarite) is the dominant transformation in the 65–110 °C range. This conclusion is consistent with the XRD results presented in Figure 1. Further evidence of the transformation dynamics is provided in Figure 4d–f, where the derivative of the conversion fraction with respect to temperature (dα/d*T*) is plotted in 3D as a function of heating rate and temperature. In Figure 4d, a distinct bell-shaped peak appears within ∼65–110 °C for the PO_4_^3–^ vibrational region with the peak temperature shifting to higher values as the heating rate increases. This peak represents the temperature (*T*_p_) of the maximum transformation rate. Notably, the conversion fraction (α_p_) at *T*_p_ remains nearly constant at ∼0.5 across all heating rates, indicating a rate-invariant conversion. This behavior suggests that the reaction mechanism associated with this vibrational region (transformation II) remains unchanged under different thermal inputs. Figure 4e captures the higher-temperature PO_4_^3–^ transformations (110–250 °C). Here, dα/d*T* does not have a well-defined peak, indicating that the reaction does not reach completion under the maximum temperature of 250 °C and is expected to be completed closer to ∼300–350 °C. In Figure 4f, a peak is observed across all heating rates for the H_2_O/NH_4_^+^ vibrational region, consistent with that detected in the PO_4_^3–^ region during transformation II. The synchronized behavior across two vibrational regions in the 65–110 °C range confirms that the struvite-to-dittmarite transformation involves simultaneous changes in both phosphate and hydrogen-bonding environments. This coupling reinforces that the process is not merely the loss of water but rather a coordinated lattice-level rearrangement affecting both PO_4_^3–^ bonds (thus symmetry) and H_2_O/NH_4_^+^ interactions.

### 3.4 Quantification of the Reaction Kinetics

To evaluate the thermal behavior of magnesium ammonium phosphate conversion into dittmarite and subsequent amorphous phases, as indicated by the ATR-FTIR results, a detailed kinetic investigation was conducted by using two complementary kinetic methods. The first was the Kissinger method, and the second was the Kissinger–Akahira–Sunose (KAS) isoconversional method.^54,55^ The methods were selectively applied to different temperature regions based on the transformation characteristics observed in the ATR-derived conversion profiles and their corresponding derivatives.

The Kissinger method is suitable for analyzing well-defined transformation peaks where the derivative of conversion fraction with respect to temperature (dα/d*T*) exhibits a clear maximum, and the second derivative is zero, as described in eq 10. It relates the heating rate (β) to the peak temperature (*T*_p_) through eq 12.^55^ Accordingly, this method was applied to transformation II (65:110 °C), where distinct *T*_p_ values were observed. As shown in Figure 5a,b, both the PO_4_^3–^ and H_2_O/NH_4_^+^ vibrational regions yielded comparable activation energies of 129.3 ± 17.9 and 126.3 ± 16.8 kJ/mol, respectively. This close agreement confirms that both vibrational domains are coupled within this temperature range and respond to approximately the same thermal energy requirement. In addition, these vibrational regions were evaluated using the KAS isoconversional method.^31^ This approach estimates the activation energy without requiring a defined dα/d*T* peak by substituting *T*_p_ with *T*_α_. Yu et al. studied struvite precipitated with NaOH/Mg­(OH)_2_ and observed activation energies ranging from 56.6 to 151.0 kJ/mol.^56^ For K-struvite, Zhang et al. estimated ∼105.9 kJ/mol assuming a single-step mechanism.^19^ Polat and Eral studied struvite with hyaluronic acid and reported an average *E*_a_ of 49.2 ± 5.1 kJ/mol by the Friedman method.^57^ In the present work, KAS analysis applied to transformation II yielded *E*_a_ values of 143.9 ± 5.5 and 140.1 ± 10.9 kJ/mol for both the PO_4_^3–^ and H_2_O/NH_4_^+^ vibrational regions, respectively. These values are based on the average activation energies of α = 0.2, 0.4, 0.6, and 0.8 which yielded values between 132.0 ± 6.1 and 162.0 ± 16.3 kJ/mol. However, further investigation is needed to clarify the specific physical interpretation of the activation energies derived from vibrational spectroscopy.

**Figure 5:**
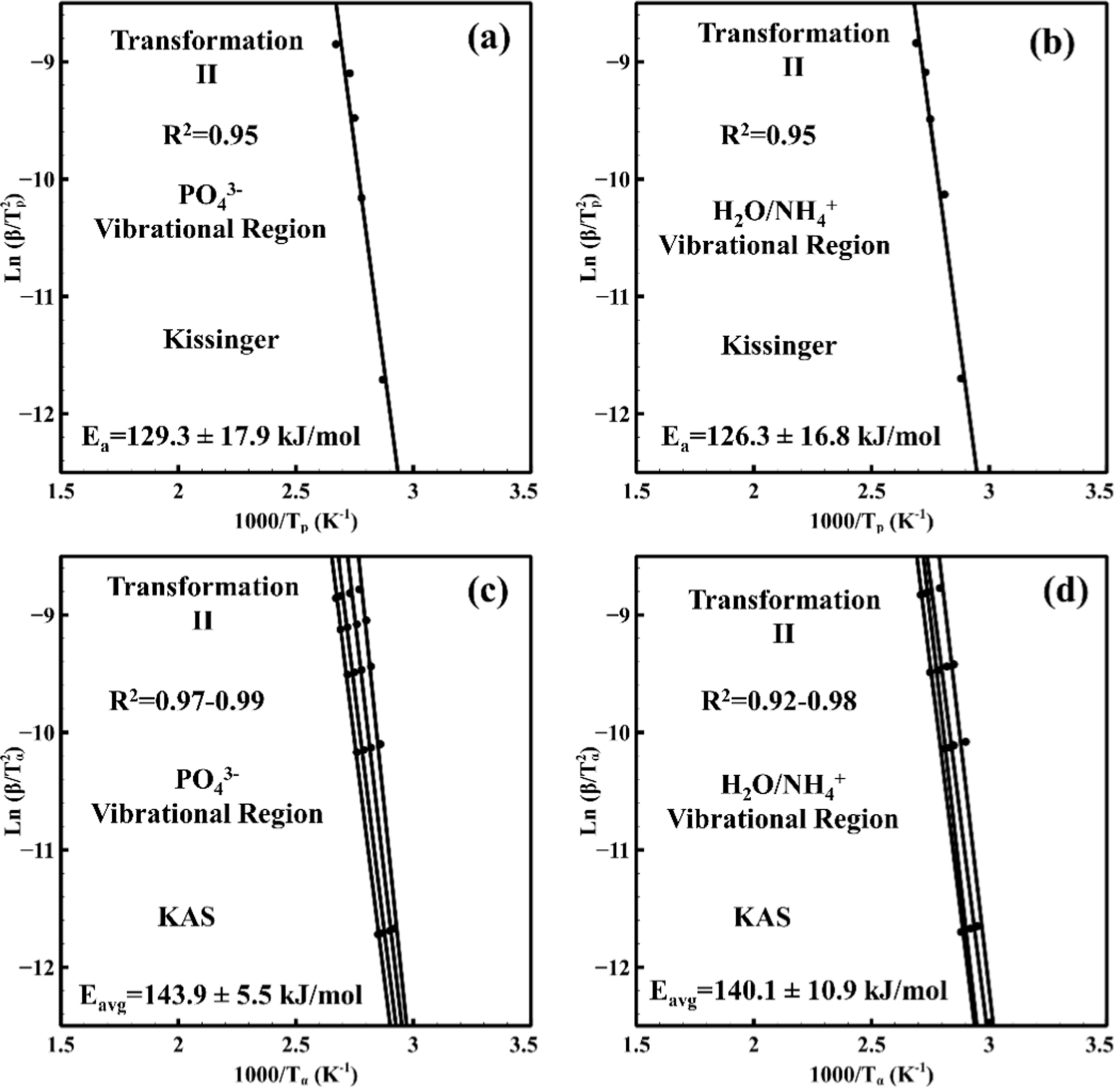
Nonisothermal in situ ATR-FTIR kinetics of transformations II in the range of 70–125 °C from (a) PO_4_^3–^ vibrational region and (b) H_2_O/NH_4_^+^ vibrational region using the Kissinger and KAS methods respectively, and (c,d) PO_4_^3–^ and H_2_O/NH_4_^+^ vibrational regions, respectively, via Kissinger and KAS methods, respectively.

### 3.5 TGA/DSC Analysis

Complementary insight into the thermal decomposition behavior of magnesium ammonium phosphate hydrates was obtained from thermal analysis, as illustrated in Figure 6. The DSC curves reveal three distinct endothermic events, while the corresponding TGA shows a total mass loss of ∼49 wt %, in strong agreement with previously reported dehydration pathways for struvite-type materials.^58^ Theoretically, one mol of H_2_O accounts for ∼7.34% mass loss; thus, loss of five coordinated waters contributes ∼36.70%. A total loss of six water molecules corresponds to ∼44.05%, and removal of NH_3_ (∼6.94%) results in a combined theoretical mass loss of ∼50.99%.

**Figure 6:**
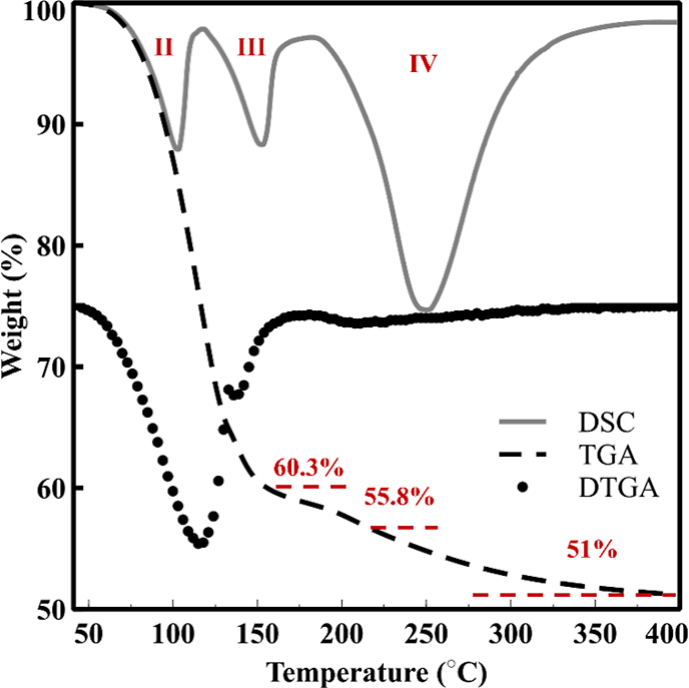
TGA, DTGA, and DSC curves of magnesium ammonium phosphate hydrate under nonisothermal heating at 10 °C/min.

The first distinct endotherm (transformation II) occurs at ∼75–130 °C and correlates well with the struvite-to-dittmarite transformation,^6,59^ involving loss of five coordinated water molecules and formation of a new crystalline structure confirmed by XRD. The remaining mass after this step (∼60.3%) corresponds to ∼39.7% loss, matching the expected stoichiometric dehydration to dittmarite.^6^ This kinetically well-defined transition is independently captured in both the PO_4_^3–^ and H_2_O/NH_4_^+^ vibrational regions. The second thermal event (transformation III), appearing near 155 °C, marks the onset of framework destabilization and partial release of the final structural water and ammonium (as NH_3_ gas). It might also belong to the amorphization of the initial dittmarite structure. The remaining mass (56.7%) aligns with the loss of six water molecules. The small amount of pre-existing dittmarite inferred from XRD (Figure 1c) suggests that this final coordinated water may originate from the initial dittmarite fraction rather than from the struvite-derived phase. The final, broad endotherm (transformation IV) spanning ∼200–350 °C corresponds to the amorphization process, culminating in the collapse of the dittmarite lattice into amorphous magnesium phosphate and removal of the remaining volatile species. This transformation is only partially observed in ATR-FTIR due to the ∼250 °C limit of the heating stage.

### 3.6 Ex Situ Raman Spectroscopy and Hyperspectral Mapping

The ex situ Raman spectra and corresponding optical microscopy images of magnesium ammonium phosphate single particles in Figure 7 illustrate the thermal evolution from 25 to 250 °C. At room temperature (Figure 7a), the Raman spectrum displays characteristic bands including the PO_4_^3–^ symmetric stretch (*v*_1_ at ∼945 cm^–1^), along with NH_4_^+^ and H_2_O vibrational features in the 1400–1700 cm^–1^ and 2800–3600 cm^–1^ regions,^51^ confirming a fully hydrated crystalline structure. The associated optical image shows a well-faceted single crystal with smooth edges. Upon heating to 100 °C, the particle largely retains its morphology with only minor edge softening, accompanied by a decrease in the NH_4_^+^ band intensity. At 150 °C, the crystal exhibits visible distortion, rounded features, and diminished reflectance, indicating the onset of structural degradation. At 250 °C, the particle has lost its crystalline integrity entirely, transitioning into a porous, mesh-like morphology with poor optical reflectance. Correspondingly, the Raman spectrum becomes dominated by broad fluorescence and shows only a weak, broadened *v*_1_ (PO_4_^3–^) band near 980 cm^–1^.^46^ This confirms substantial amorphization driven by the release of coordinated water and ammonium, leading to the formation of defects throughout the structure.

**Figure 7:**
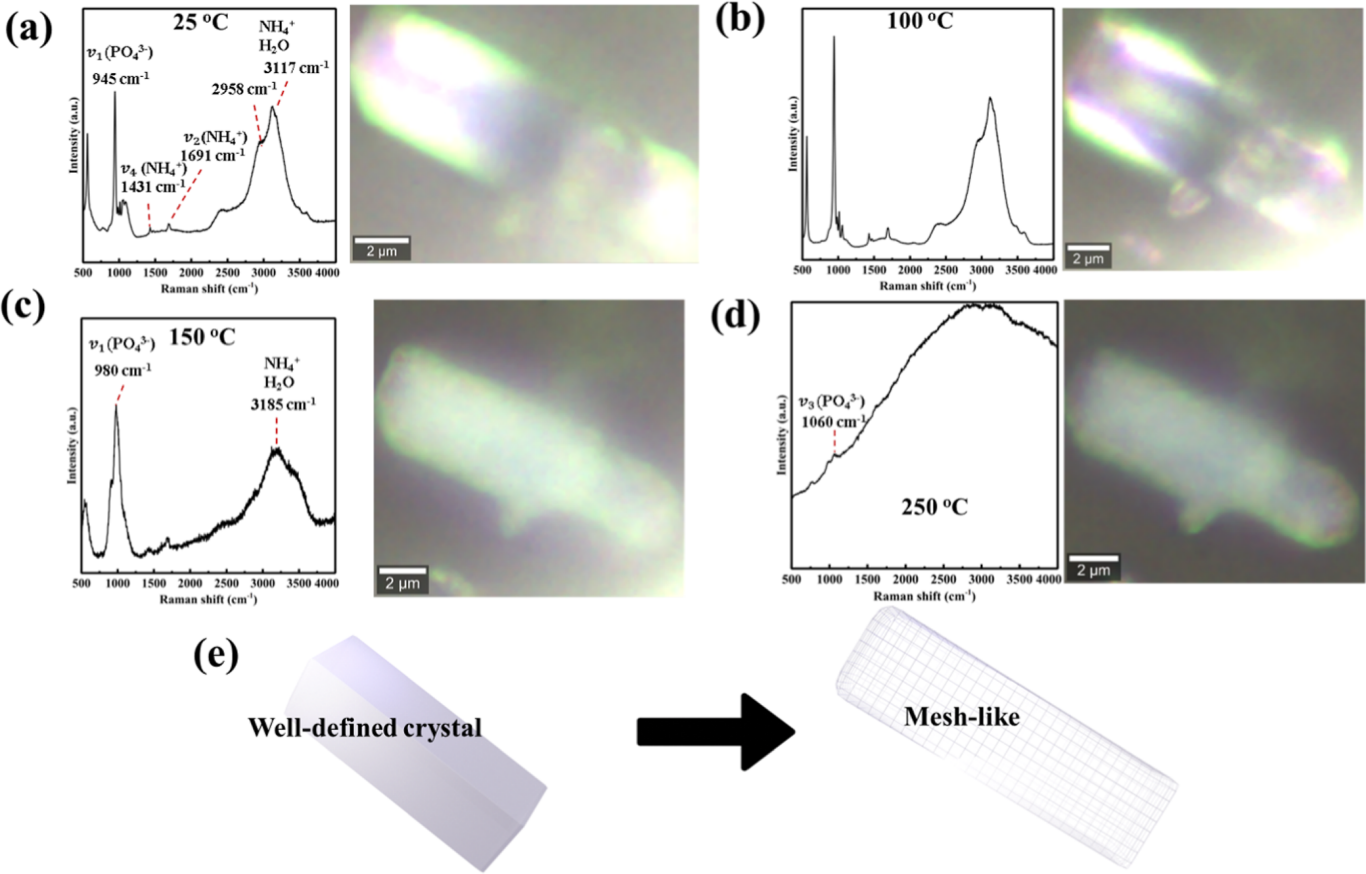
Temperature-resolved ex situ Raman spectromicroscopy of an individual struvite particle at 25 (a), 100 (b), 150 (c), and 250 °C (d). The micrographs paired with spectra to the changes in bands alongside the morphology evolution. (e) An illustration for the morphology transformation from the well-defined crystal to the mesh-like morphology.

The thermally resolved hyperspectral Raman mapping presented in Figure 8 shows the temperature-dependent phase evolution of hydrated magnesium phosphate particles. Total Component Analysis (TCA) is applied to distinguish spectral entities represented by using color-coded phase assignments. At room temperature (Figure 8a), a dominant TCA component is observed and assigned to struvite (blue), supported by ex situ XRD and Raman results. Its associated spectrum exhibits strong PO_4_^3–^ (945 cm^–1^), NH_4_^+^ (1431 and 1691 cm^–1^), and H_2_O (3117 cm^–1^) vibrations. Minor green regions correspond to newberyite, confirmed by its characteristic spectral features including a nondegenerate PO_4_^3–^ region (892, 980, and 1050 cm^–1^) associated with lower HPO_4_^2–^ symmetry and the presence of lattice P–OH modes.^13^ As the temperature increases to 100 °C (Figure 8b), a major transformation occurs, with widespread formation of a component assigned to dittmarite (red), accompanied by a decreasing fraction of untransformed struvite and persistence of trace newberyite. At 150 °C (Figure 8c), dittmarite becomes the dominant phase, with only isolated regions exhibiting amorphous characteristics (broad featureless PO_4_^3–^ profiles). Dittmarite is identified by PO_4_^3–^ modes at 905 and 980 cm^–1^ and NH_4_^+^ features at 1431 and 1691 cm^–1^, distinguishing it from newberyite, which has no ammonium ions in its lattice. At 250 °C (Figure 8d), the TCA indicates predominantly amorphous material (magenta), with only minor remnants of crystalline dittmarite and struvite. This progression is corroborated by the spectra in Figure 8e, which show increasingly broader and weakened Raman bands at higher temperatures, consistent with structural collapse and amorphization driven by the loss of coordinated water and ammonium.

**Figure 8:**
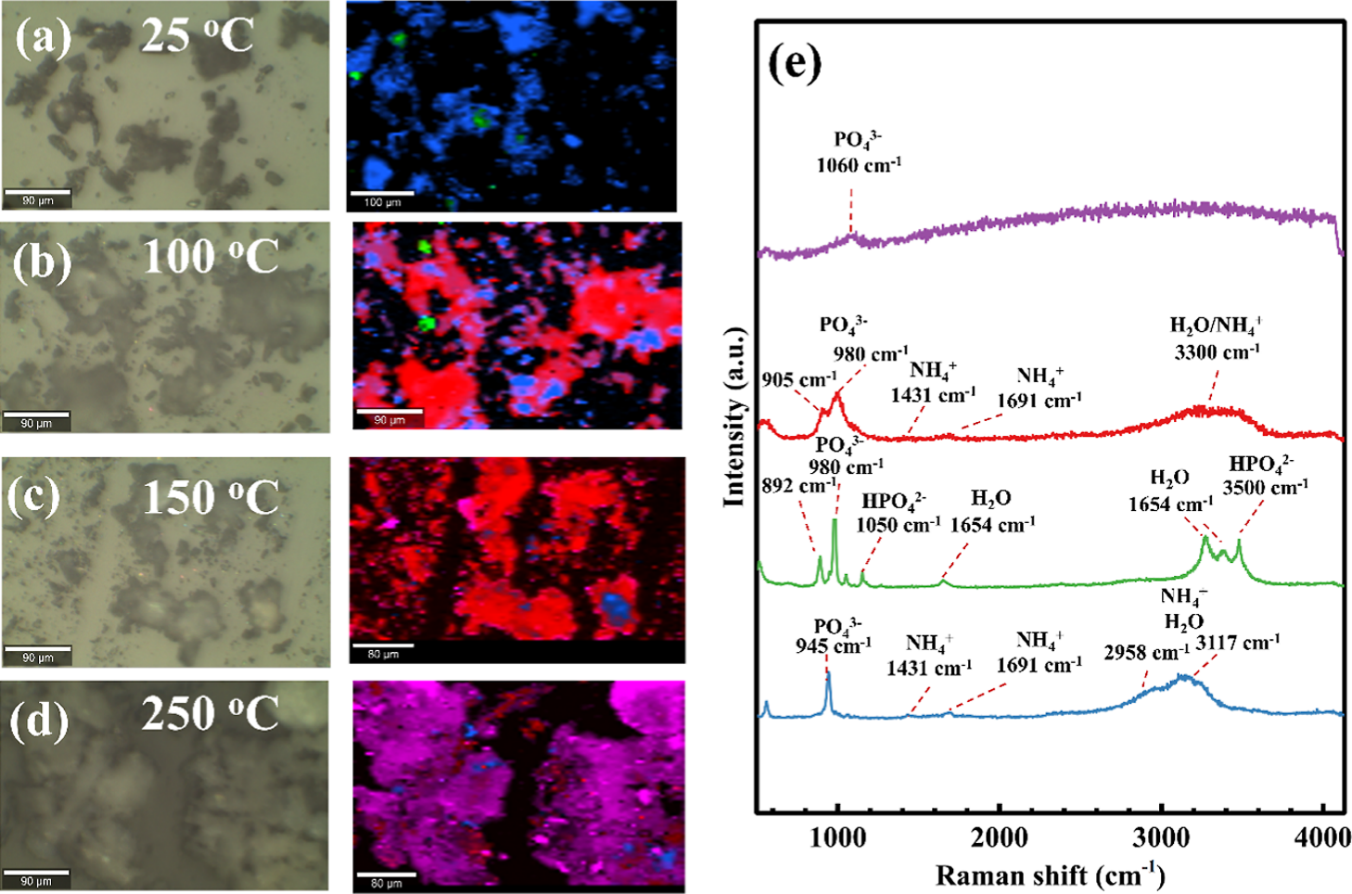
(a–d) Optical micrographs and the corresponding Raman TCA maps of magnesium ammonium phosphate hydrate acquired at different temperatures with the corresponding spectra shown in (e).

## 4 Conclusions and Environmental Relevance

This work developed and applied an LLS spectral processing approach for determining nonisothermal kinetics of magnesium ammonium phosphate hydrate decomposition using in situ ATR-FTIR spectroscopy. The method successfully correlates vibrational, structural, and compositional changes through quantitative spectral deconvolution. Figure 9 shows four distinct transformation regimes identified: (i) desorption of weakly bound water below ∼65 °C, (ii) crystallographic dehydration and lattice reorganization during the primary struvite-to-dittmarite transformation (65–110 °C), and continued dehydration and amorphization (iii) above ∼110 °C for pre-existing dittmarite in the starting material and (iv) above ∼150 °C for the dittmarite formed from struvite.

**Figure 9:**
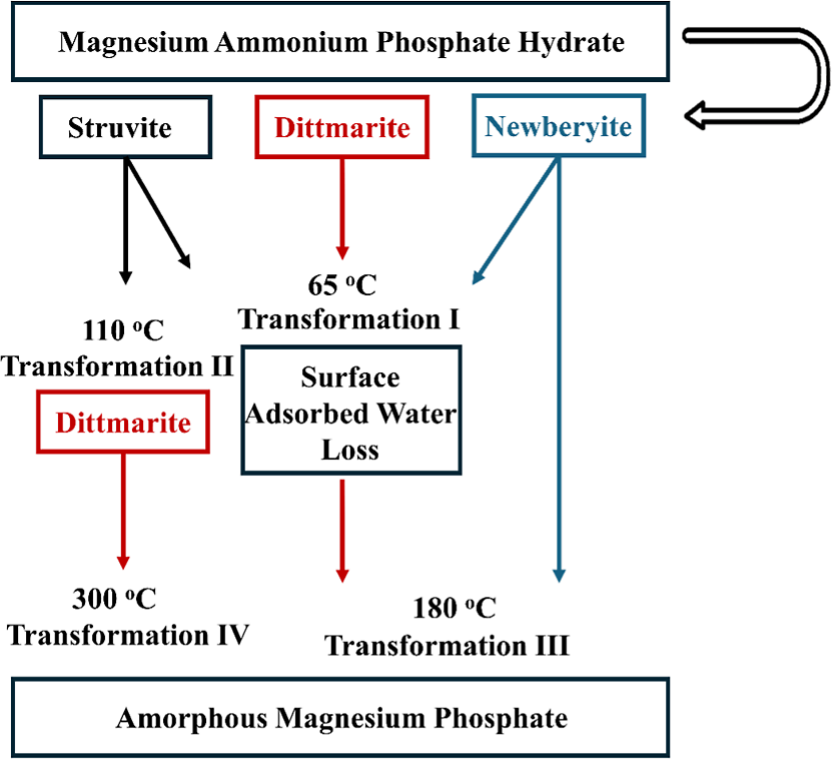
Schematic diagram for the detected transformation of magnesium ammonium phosphate hydrate under heating conditions in air.

Complementary in situ XRD and ex situ Raman imaging confirmed the structural evolution and the phase-stability sequence. Collectively, these results demonstrate that in situ ATR-FTIR, when processed through an LLS model, provides a quantitative surface-sensitive tool that complements bulk thermogravimetric techniques for kinetic studies of hydrated magnesium phosphate transformations. A noteworthy observation requiring further study is the temperature offset between events detected by ATR-FTIR and those observed in DSC/TGA. This discrepancy likely arises from the fundamental differences in measurement modalities. Furthermore, the physical meaning is where DSC/TGA captures bulk thermochemical and mass-loss changes, whereas ATR-FTIR detects local structural and bonding rearrangements near the sample surface. As a result, dehydration, phase transformation, or reorganization of phosphate and ammonium groups may be detected at earlier stages spectroscopically than in bulk thermal signatures (with different activation energies). Understanding and quantifying this might refine kinetic correlations, improve activation-energy determinations, and strengthen the link between surface-specific conversion and bulk decomposition behavior.

## Data Availability

All data supporting the findings of this study are available from the corresponding author upon reasonable request.
